# Mechanical Treatment of Vomiting in Pregnancy

**Published:** 1888-02

**Authors:** J. W. Flanders

**Affiliations:** Wrightsville, Ga.


### MECHANICAL TREATMENT OF VOMITING IN PREGNANCY.*

BY J. W. FLANDERS, M. D., WRIGHTSVILLE, GA.

Nausea and vomiting in pregnancy is so common that it is regarded as a physiological necessity, and a majority get well without treatment. There are cases, however, which resist all treatment short of the complete removal of the cause. The belief by the people that vomiting is absolutely necessary for the well-being of pregnant women has no doubt caused a great deal of suffering where relief might have been obtained. I have often heard great fears expressed for pregnant women on account of the absence of nausea and vomiting, and for this reason we are seldom called on to treat those cases until their conditions have become alarming.

*Read before the Medical Association of Georgia. From the Transactions.

In 1875 a physician in Baltimore, whose name I have forgotten, published in the *Maryland Medical Journal* his experience in the treatment of one of those uncontrollable cases. He was called into the country to see a lady in her first pregnancy, suffering from nausea and vomiting, and after exhausting his skill in the vain attempt to relieve her, and seeing that she could not live much longer without relief, suggested to her husband that he would have to produce abortion or his wife would die. He urged the doctor to do anything that gave any hopes of relief. He attempted to produce abortion by pushing his finger through the cervix and rupturing the membranes. Failing in this he returned to the city to procure assistance, and to his astonishment, when he returned, found his patient quiet, not having vomited since he left her. He did nothing more for her, and she went to term without further trouble.

I see in Pepper's System of Medicine that Dr. Copeman, of Norwich, England, claims to have made the same discovery of this plan of treatment about the same time and in the same way. It is a little strange that two men should have relieved a patient about the same time, in the same way precisely, under the same circumstances, and unintentionally at that.

Not long after the publication of the article referred to in the *Maryland Medical Journal*, I was hastily summoned to see Mrs. S., who was pregnant for the first time. Realizing the fact that her case was a grave one, I lost no time in getting to her bedside. She had been vomiting several days—in fact, had retained nothing on her stomach for a week. Her extremities were cold and pulse not strong enough to be felt at the wrist, and she was in a state of low, muttering delirium and vomiting a glairy mucus constantly. I gave her a dose of oxalate of cerium and ingluvin combined, and oiled my index finger and gently worked it into the cervix and pushed the uterus up. The vomiting ceased immediately. In a few minutes wine was administered, which was retained, it being the first thing her stomach had retained in a week. She went to term without further trouble; however, she was instructed to take a dose of lactopeptine after each meal.

Soon after this a medical friend, living in an adjoining county, called to consult me about the case of a lady to whom he had been called a few days before, and stated that she had been vomiting about four weeks, and during all this time had retained nothing on her stomach. She was then reduced to a skeleton, and was so weak that she could not turn her head on the pillow without assistance. She had been under the treatment of another physician who had abandoned her to her fate. She was still vomiting a thin, glairy mucus. I suggested to him the mechanical treatment above described. He reported to me that he carried out my suggestions, and she vomited only once afterward, and he thought that was caused by some error in diet, and in a remarkably short time she had so far recovered as to attend to her household duties.

I could give other cases in which this treatment was successful, but deem it unnecessary. There have been so many articles published in THE MEDICAL JOURNAL within the last few years upon this plan of treatment that it seems useless to bring it to the notice of the profession again, but it does seem to me that any suggestion which gives promise of aid in this distressing class of cases would naturally attract attention, and should be given an opportunity at least to prove its right and title to a place on the list.

If it is true that increased functional activity of the spinal cord and nervous system is the cause of vomiting in pregnancy, as maintained by most authors, I cannot explain how this course of treatment gives relief, but I think Graily Hewitt is right in maintaining that uterine displacements are the most potent causes of vomiting in pregnancy.

